# Acute Variceal Bleeding During the SARS-CoV-2 Pandemic: A National Multicenter Observational Study

**DOI:** 10.3390/jcm14176166

**Published:** 2025-08-31

**Authors:** Gabriel Allo, Stefanie Quickert, Karsten Große, Sidar Baysal, Dirk Nierhoff, Christoph Neumann-Haefelin, Christoph Schramm, Tony Bruns, Philipp Alexander Reuken, Martin Bürger

**Affiliations:** 1 Department for Gastroenterology and Hepatology, Faculty of Medicine, University Hospital of Cologne, University of Cologne, 50937 Cologne, Germany; 2 Department of Internal Medicine IV (Gastroenterology, Hepatology and Infectious Diseases), Jena University Hospital, 07747 Jena, Germany; 3 Department of Internal Medicine III, University Hospital RWTH Aachen, 52074 Aachen, Germany; 4 Department of Gastroenterology, Hepatology and Transplant Medicine, Medical Faculty, University of Duisburg-Essen, 45147 Essen, Germany

**Keywords:** upper gastrointestinal bleeding, cirrhosis, portal hypertension, COVID-19, pandemic, acute variceal bleeding

## Abstract

**Background**: The COVID-19 pandemic disrupted healthcare systems globally, raising concerns about its negative impact on patients with chronic liver diseases by contributing to hepatic decompensations such as acute variceal bleeding (AVB). This study aimed to evaluate the impact of the COVID-19 pandemic on clinical outcomes in cirrhotic patients with AVB in Germany. **Methods**: This retrospective national multicenter study compared patients with cirrhosis and AVB treated at four tertiary care centers in Germany before (2016–2020) and during the pandemic (2020–2022). The primary endpoint was 6-week mortality, and secondary outcomes included infections, transfusion requirement and rebleeding. **Results**: The baseline characteristics of the 247 patients were largely comparable between the two groups, however metabolic dysfunction-associated steatotic liver disease was more prevalent during the pandemic compared to the pre-pandemic period (12.5% vs. 4.8%, *p* = 0.048). Only one patient tested positive for SARS-CoV-2. Six-week mortality (32.2% vs. 30.1%; *p* = 0.767) and rebleeding rates (22.8% vs. 22.3%; *p* = 1.000) did not differ significantly between groups. Interestingly, intubation rates, length of stay on the intensive care unit, post AVB infection rates and types of infection were also comparable (all *p* > 0.05), while transjugular intrahepatic portosystemic shunt placement (TIPS) after bleeding was performed more frequently during the pandemic (23.2% vs. 11.3%, *p* = 0.019). **Conclusions**: Relevant patient-related AVB outcomes were unaffected during the COVID-19 pandemic. These findings suggest the resilience of critical AVB management practices in German tertiary centers. The increased use of TIPS and MASLD prevalence during the pandemic may reflect evolving clinical practice and patient profiles warranting further investigation.

## 1. Introduction

The coronavirus disease 2019 (COVID-19) pandemic led to unprecedented challenges in nearly all aspects of human life, particularly in global healthcare systems. The urgent allocation of medical resources to COVID-19 patients and the implementation of preventive measures such as social distancing significantly impacted the accessibility of standard care for non-COVID-19 patients, including those with chronic diseases [1]. Health practitioners were forced to reduce or postpone regular appointments, elective procedures like endoscopic band ligations, and hepatitis therapies, making patients with chronic liver disease (CLD), especially with advanced chronic liver disease, particularly vulnerable [2,3]. Patients with cirrhosis, and even more so in the presence of hepatic decompensation, are threatened by a high risk of mortality from COVID-19, which is largely determined by cirrhosis-associated comorbidities and extrahepatic organ failure [4]. In particular, patients who presented with decompensated liver cirrhosis had a significantly higher mortality rate in the event of a COVID-19 infection [5]. In the United States, there was a significant increase in national CLD and cirrhosis-related mortality during the COVID-19 pandemic, and age-standardized mortality rates for alcohol-associated liver disease and metabolic dysfunction-associated steatotic liver disease (MASLD) increased at an alarming rate during the COVID-19 pandemic, with the largest disparities among the young, non-Hispanic White, and Alaska Indian/Native American populations [6,7]. Furthermore, cirrhosis itself is an independent predictor for COVID-19 mortality [8]. Moreover, a significant increase of alcohol consumption during the pandemic among high-risk alcohol users led to a growing concern for a long-term increase in the burden of CLD-related complications such as decompensations and acute variceal bleeding (AVB) [9]. Additionally, protecting healthcare personnel was a critical priority during the pandemic, as frontline workers faced an elevated risk of morbidity and mortality due to direct exposure to SARS-CoV-2 [10]. Ensuring adequate infection control protocols while maintaining essential services for patients with acute complications such as AVB posed a significant challenge [11,12].

Recent studies have demonstrated an overall reduction in emergency endoscopic procedures for non-variceal upper gastrointestinal bleeding (UGIB) during the initial waves of the pandemic [13], while the amount of endoscopic procedures for AVB remained relatively stable [12]. Despite increased rates of active bleeding detected during endoscopy [14], the impact of COVID-19 on patient outcomes, such as transfusion requirements and survival, remains inconclusive. While some reports suggest worse outcomes due to limited healthcare access [15], others indicate comparable survival rates between COVID-19-infected and non-infected patients [16].

Given the insufficient evidence regarding AVB, further research is necessary to clarify the pandemic’s influence on this patient population.

The primary aim of this study was to analyze the impact of the COVID-19 pandemic on key patient-related outcomes in the setting of AVB. By comparing pre-pandemic and pandemic cohorts, we sought to evaluate whether healthcare system adaptations affected patient outcomes such as mortality, rebleeding and infection rates.

## 2. Materials and Methods

In this retrospective national multicenter study, we compared baseline characteristics and outcomes of patients with cirrhosis and AVB, who were treated at four tertiary care centers in Germany (University Hospitals of Cologne, Aachen, Jena, and Essen) before (1 March 2016–28 February 2020; 1 March 2018–28 February 2020 for the University Hospital of Essen) and during the COVID-19 pandemic (1 March 2020–1 March 2022). Inclusion criteria were age above 18 years, presence of cirrhosis and AVB. Exclusion criteria included death before endoscopy as well as initial endoscopy or subsequent treatment following endoscopy at another hospital.

### 2.1. Endpoints and Definitions

The primary outcome was 6-week mortality after index bleeding. Secondary outcomes included rebleeding, radiological or surgical intervention to control bleeding, intensive care unit admission, number of packed red blood cells transfused, rebleeding, bacterial infections after the bleeding event and length of stay.

Cirrhosis was diagnosed by histological criteria or by typical clinical, biochemical and imaging findings. AVB was assumed when clinical signs of UGIB (hematemesis, melena or hematochezia) were present and confirmed by esophagogastroduodenoscopy (EGD). Furthermore, when bleeding stigmata were absent during endoscopy, AVB was only diagnosed when no other possible bleeding source than varices was observed [17]. Rebleeding was defined as further hematemesis, passage of fresh melena after initial normalization of the stool or recurrent endoscopic stigmata of AVB during follow-up EGD. Bacterial infection and multi-drug-resistance (MDR) were diagnosed by clinical, radiological and laboratory findings and were based on conventional criteria [18,19]. Spontaneous bacterial peritonitis was defined according current guidelines [20].

### 2.2. Treatment

Patients with AVB were treated in accordance with the prevailing clinical standards and continuously updated guidelines as applicable during the respective treatment period [20,21]. Emergency EGD was performed by on-call teams of experienced physicians on a 24/7 basis. When diagnosed endoscopically, bleeding esophageal varices were treated with band ligation during the same procedure. Vasopressors were started when AVB was assumed and continued for up to five days. Additionally, all patients received antibiotic prophylaxis for at least five days. Rescue TIPS was performed in the case of uncontrollable bleeding, while preemptive TIPS was placed according to current guidelines [22].

### 2.3. Data Collection

The following data were retrieved from the medical records of included patients: age, gender, etiology and clinical signs of liver cirrhosis (ascites, hepatic encephalopathy), Child-Turcotte-Pugh score (CTP), model of end-stage liver disease (MELD) score, arterial blood pressure, heart rate, signs and symptoms of AVB, comorbidities in accordance with the Charlson comorbidity index (CCI) [23], laboratory values at presentation (lactate, hemoglobin, albumin, creatinine, bilirubin, prothrombin time/International Normalized Ratio (INR), thrombocyte count), endoscopic findings, antibiotic prophylaxis, occurrence of infection after bleeding, identified pathogens, endoscopic, radiological and surgical interventions for hemostasis, liver transplantation during the hospital stay or within 42 days after AVB, discharge date, number of blood transfusions, rebleeding and death after 42 days.

### 2.4. Statistical Analysis

Descriptive analysis was conducted using the Statistical Package for the Social Sciences (SPSS), version 28 (IBM, Armonk, NY, USA). Categorical variables were presented as absolute, and relative frequencies and continuous variables were expressed as median with interquartile range (IQR). *p*-values < 0.05 were regarded as statistically significant.

### 2.5. Ethical Approval

This study was performed in accordance with the Declaration of Helsinki and approved by the institutional ethical review board (23-1347-retro). Written, informed patient consent was waived due to the strict retrospective design of this study.

This article is a revised and expanded version of a paper entitled ACUTE VARICEAL BLEEDING DURING THE SARS-CoV-2 PANDEMIC (PP0984), which was presented at UEG Week 2024, Vienna, Austria, 12–15 October 2024 [24].

## 3. Results

**Figure 1:**
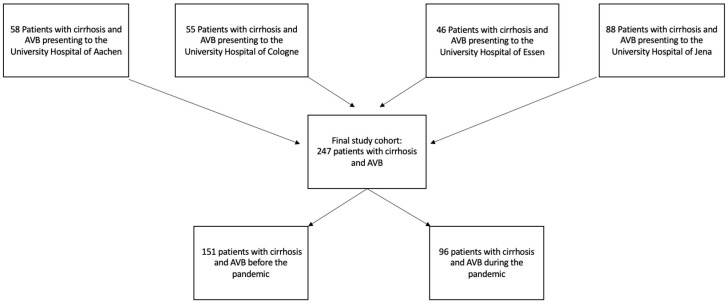
Study flow chart. Abbreviations: AVB: acute variceal bleeding.

Over the study period, 247 cases of AVB were identified, 151 in the pre-pandemic era and 96 during the pandemic (Table 1 and Figure 1). The population included 179 (73.4%) men, with a median age of 57 years (IQR 49–64 years). Alcohol was the leading cause of liver cirrhosis in 131 (54.1%) cases, and 97 patients (42.2%) reported ongoing alcohol consumption. The leading clinical sign of bleeding was hematemesis in 165 cases (67.1%), followed by melena in 110 (44.9%), with 113 (52.3%) patients presenting with signs of shock. Baseline characteristics did not differ significantly between the two groups, besides MASLD occurring more frequently during the pandemic (4.8% vs. 12.5%; *p* = 0.048). Furthermore, median albumin levels appeared to be higher during the pandemic (25 [IQR 21–30] vs. 28 [IQR 24–33]; *p* < 0.001), while thrombocyte counts (124 [IQR 71–181] vs. 102 [IQR 56–145]; *p* = 0.014) and prothrombin time levels (50 [IQR 40–66] vs. 45 [IQR 30–58]; *p* = 0.027) were lower. Additionally, we noticed a non-significant trend towards higher median bilirubin levels (2 [IQR 1.0–4.1] vs. 2.7 [IQR 1.3–4.5]; *p* = 0.061) and a higher median MELD score during the pandemic (16 [IQR 12–22] vs. 19 [IQR 12–26]; *p* = 0.06).

Active bleeding was detected in 138 (55.9%) cases, while hemostasis at the end of endoscopy was successful in 112 of 128 documented cases (87.5%) (Table 2). A total of 196 (79.7%) patients were treated at the intensive care unit, with a median length of stay of three days (IQR 2–8 days). Rebleeding within six weeks occurred in 54 (22.5%) cases, and the six-week mortality rate was 30.9%. Endoscopic bleeding signs as well as analyzed bleeding management and outcomes did not differ between the two periods, though TIPS placement was performed more frequently during the pandemic (11.3% vs. 23.2%; *p* = 0.019).

Infections after AVB were detected in 73 (30.3%) patients (Table 3). Bacterial pathogens were detected in 42 (17.3%) cases, with 17 (6.9%) involving MDR bacteria. In these cases, pneumonia was the most frequent type of infection (n = 27, 37.0%), followed by spontaneous bacterial peritonitis (n = 11, 15.1%) and primary bacteriemia (n = 11, 15.1%). Infection characteristics and intubation rates did not differ between the two periods. Only one patient (1.0%) tested positive for SARS-CoV-2 during their hospital stay, while two other patients (2.0%) had a history of infection. The rates of spontaneous bacterial peritonitis after AVB did not differ between patients with and without ascites at bleeding events (6.4% vs. 2.1%; *p* = 0.207).

## 4. Discussion

Our study provides valuable insights into the impact of the COVID-19 pandemic on patients with AVB and their clinical outcomes in Germany. Despite concerns regarding restricted healthcare access and resource allocation, our findings suggest that key patient-related outcomes, including six-week mortality and infection rates, remained largely unchanged between the pre-pandemic and pandemic periods, suggesting that the direct and indirect effects of the pandemic had no significant impact on the outcome of patients with AVB in Germany.

Gao et al. found, in their study, reported an increase in UGIB-related mortality during the COVID-19 pandemic [7]. These results are consistent with previous studies, which reported a decline in hospital visits due to transportation disruptions and fear of infection, potentially resulting in delayed treatment and worse outcomes. A U.S. nationwide study found that, while overall UGIB-related hospital admissions decreased by 9.5% in 2020, mortality among these patients rose by 13% [25]. Furthermore, two cohort studies found that patients with UGIB had lower hemoglobin levels compared to those treated before the pandemic [6,26]. Additionally, in a population-based study from Hong Kong, patients with UGIB had a greater need for blood transfusion, and there was a significant increase in the proportion of patients with AVB after COVID-19, which may be due to the delay in presentation of some patients with minor bleeding [26]. Likewise, another study observed a decline in emergency department visits for UGIB during the pandemic, accompanied by a higher rate of hospital admissions and more severe cases among those who did present [27]. The fear of infection, along with the implementation of policies and restrictions to limit the spread of the virus, resulted in a substantial decline in hospital visits for conditions unrelated to COVID-19 [28]. This is especially worrisome for conditions like UGIB, which require timely intervention to prevent worse outcomes.

Interestingly, in our study, while laboratory findings demonstrated some variations—such as lower thrombocyte counts and prothrombin time during the pandemic—the overall clinical characteristics of patients, including hemoglobin levels and severity scores like MELD and CTP, remained comparable across the two time periods. These findings suggest that the impact of the COVID-19 pandemic on healthcare access and resource allocation did not result in more severe cases of AVB in Germany.

Infections remain a major concern in patients with cirrhosis and UGIB, as they are associated with worse outcomes [18,29,30]. We observed that the overall infection rates, including bacterial pathogen detection and MDR organisms, did not differ significantly between the pre-pandemic and pandemic groups. This may reflect consistent adherence to prophylactic antibiotic guidelines and infection control measures during both time periods. Our findings align with a study conducted in Spain, which also demonstrated similar infection rates among hospitalized patients with cirrhosis during the two periods [31]. In contrast, the infection rates in patients with Hodgkin lymphoma undergoing chemotherapy were significantly lower during the pandemic at our tertiary center, suggesting that the intensified protective measures implemented during the COVID-19 pandemic had a beneficial effect on this patient group [32]. These differing results indicate that the implied enhanced protective measures may not be effective in reducing infection rates following UGIB in cirrhotic patients. Although speculative, this could be due to the fact that most infections following UGIB in cirrhotic patients are respiratory in nature, typically associated with aspiration during the acute bleeding event and peritonitis resulting from bacterial translocation [33]. Therefore, intensified measures aimed at preventing pathogen translocation may be ineffective in this context.

The relatively low intubation rate of 16% despite this severely ill cohort may be explained by several factors, such as effective early hemodynamic stabilization, a less pronounced clinical severity of hematemesis or hepatic encephalopathy in some patients, or a generally more conservative approach to intubation in our centers. Interestingly, intubation rates and ICU admissions did not differ significantly between the two periods. The potential concern regarding SARS-CoV-2 transmissions during intubations, as well as ICU admission triage policies during the pandemic, may not have influenced general clinical practice in the management of AVB.

The finding that only one patient tested positive for SARS-CoV-2 during hospitalization suggests that rigorous infection control protocols were effective in preventing nosocomial COVID-19 infections. The low prevalence of COVID-19 among our cohort further supports that AVB alone does not inherently increase susceptibility to SARS-CoV-2 infection.

The relatively high mortality rate of 30.9% may be explained by the advanced liver disease severity in our population, as reflected by a median CTP score of 10 and severe clinical bleedings signs, indicating a high-risk patient group.

A notable finding of our study is the increased frequency of TIPS placement during the pandemic, suggesting a possible shift toward more advanced management strategies. This may reflect an increased awareness of the importance of early preemptive TIPS placement to reduce the risk of rebleeding and mortality in high-risk patients [34].

Moreover, the higher prevalence of MASLD during the pandemic warrants further investigation, as it may indicate changes in the underlying etiology of cirrhosis due to pandemic-related lifestyle modifications [35]. In patients with MASLD, Rivera-Esteban et al. observed that while metabolic control had indeed deteriorated after the first year of the pandemic and patients showed poorer clinical outcomes, the latter was largely attributable to causes unrelated to the liver—most notably, COVID-19 itself. Regarding hepatocellular carcinoma diagnostic delay and prophylactic oesophageal varices treatment, no differences were found when comparing one year before and after the pandemic [36].

Our study has some limitations. First, its retrospective design may introduce selection bias, and the data are limited to four tertiary care centers in Germany, which may not be generalizable to other healthcare settings. Furthermore, we cannot exclude that patients in the broader region were admitted in other hospitals. However, since patients with severe liver disease in Germany are usually managed and referred to specialized liver centers, we assume that the majority of AVB cases in the catchment areas were captured in our cohort. Second, while we observed trends in infection rates and laboratory findings, statistical significance was not reached for all variables. Larger studies with greater statistical power should confirm these results. Lastly, the impact of pandemic-related healthcare system constraints, such as delayed hospital presentations or limited intensive care unit capacity, could not be fully accounted for in our analysis.

In conclusion, our findings suggest that despite the challenges posed by the COVID-19 pandemic, essential management strategies for AVB remained effective, with stable mortality and rebleeding rates in our cohort. Increased utilization of TIPS and evolving cirrhosis etiologies warrant further investigation. Future studies should aim to explore the long-term implications of pandemic-related healthcare adaptations on liver disease management and patient outcomes.

## Figures and Tables

**Table 1:** Baseline characteristics.

Variables	Total	Pre-COVID-19 Era (01.03.2016–28.02.2020)	COVID-19 Era (01.03.2020–28.02.2022)	*p*-Value
Cases (%)	247 (100)	151 (61.1)	96 (38.9)	
Men (%)	179 (73.4)	109 (72.2)	70 (75.3)	0.656
Age (years, IQR)	57 (IQR 49–64)	58 (49–65)	56 (51–62)	0.566
Clinical sign of bleeding (%)				
Hematemesis	165 (67.1)	100 (66.7)	65 (67.7)	0.89
Melena	110 (44.9)	68 (45.6)	42 (43.8)	0.794
Hematochezia	21 (8.5)	14 (9.3)	7 (7.3)	0.646
Syncope	26 (10.5)	17 (11.3)	9 (9.4)	0.677
Impaired mental status	68 (27.6)	38 (25.3)	30 (31.3)	0.381
Signs of shock	113 (52.3)	66 (51.6)	47 (53.4)	0.789
Charlson comorbidity index (IQR)	4 (3–6)	4 (3–6)	4 (3–5)	0.941
Active alcohol consumption (%)	97 (42.2)	61 (44.2)	36 (39.1)	0.496
Prior band ligation (%)	74 (34.3)	52 (37.7)	22 (28.2)	0.181
History of UGIB (%)	99 (41.3)	55 (37.3)	44 (46.8)	0.18
Medication (%)				
Aspirin	51 (21.1)	25 (17.0)	26 (27.4)	0.075
Dual antiplatelet therapy	10 (4.2)	6 (4.2)	4 (4.3)	1.000
anticoagulants	25 (10.6)	16 (11.2)	9 (9.7)	0.705
Non-selective beta-blockers	107 (45.0)	61 (42.4)	46 (48.9)	0.352
Etiology of cirrhosis (%)				
Alcohol-associated	131 (54.1)	85 (58.2)	46 (47.9)	0.147
Hepatitis C	19 (7.9)	13 (8.9)	6 (6.3)	0.626
Hepatitis B	8 (3.3)	3 (2.1)	5 (5.2)	0.271
MASLD	19 (7.9)	7 (4.8)	12 (12.5)	0.048
Autoimmune Hepatitis	7 (2.9)	3 (2.1)	4 (4.2)	0.44
PSC	7 (2.9)	4 (2.8)	3 (2.9)	0.684
PBC	6 (2.5)	2 (1.4)	4 (1.7)	0.218
Unknown	22 (9.1)	16 (11.0)	6 (6.3)	0.258
Laboratory (IQR)				
Lactate [mmol/L] (n = 215)	2.8 (1.7–4.7)	2.8 (1.7–4.7)	2.8 (1.7–4.5)	0.817
Albumin [g/L] (n = 236)	26 (22–31)	25 (21–30)	28 (24–33)	<0.001
Creatinine [mg/dL] (n = 245)	1.1 (0.8–1.6)	1.0 (0.8–1.6)	1.2 (0.8–1.8)	0.117
Bilirubin [mg/dL] (n = 244)	2.3 (1.2–4.3)	2 (1.0–4.1)	2.7 (1.3–4.5)	0.061
White blood cell count [×10^9^/L]	10.2 (7.1–15.7)	10.4 (7.3–17.0)	10.0 (7.0–15.4)	0.507
Hemoglobin [g/dL]	7.9 (6.8–9.7)	8 (6.7–9.8)	7.9 (6.9–9.5)	0.892
Thrombocytes [×10^9^/L]	114 (68–170)	124 (71–181)	102 (56–145)	0.014
Prothrombin time [%]	48 (36–64)	50 (40–66)	45 (30–58)	0.027
Ascites (according to CTP)				0.943
Mild	72 (30.1)	44 (30.8)	28 (29.2)	
Severe	72 (30.1)	42 (29.4)	30 (31.3)	
CTP (n = 216)	10 (8–12)	10 (8–11)	10 (8–12)	0.328
MELD (n = 236)	16 (13–23)	16 (12–22)	19 (12–26)	0.06

Abbreviations: CTP: Child Pugh Turcotte score; MASLD: metabolic disfunction-associated steatotic liver disease; MELD: measurement of end-stage liver disease, PBC: primary biliary cirrhosis; PSC: primary sclerosing cholangitis.

**Table 2:** Outcome.

	Total	Pre-COVID-19 Era (01.03.2016–28.02.2020)	COVID-19 Era (01.03.2020–28.02.2022)	*p*-Value
Blood transfusion (%)	171 (70.7)	110 (74.3)	61 (64.9)	0.147
TIPS after bleeding (%)	39 (15.9)	17 (11.3)	22 (23.2)	0.019
Rescue	6 (15.4)	3 (17.6)	3 (13.6)	1.000
Preemptive	18 (46.2)	8 (47.1)	10 (45.5)	1.000
Other indication	14 (35.9)	6 (35.3)	8 (36.4)	1.000
Surgery for hemostasis (%)	2 (1.1)	0	2 (2.6)	0.163
Liver transplantation after bleeding (%)	4 (1.6)	1 (0.7)	3 (3.1)	0.302
Length of stay on the ICU (days) n = 209, IQR	3 (2–8)	3 (2–8)	4 (2–8)	0.917
Rebleeding within six weeks (%)	54 (22.5)	33 (22.3)	21 (22.8)	1.000
Death within six weeks (%)	68 (30.9)	40 (30.1)	28 (32.2)	0.767

Abbreviations: ICU: intensive care unit; TIPS: transjugular intrahepatic portosystemic shunt.

**Table 3:** Infection characteristics.

	Total	Pre-COVID-19 Era (01.03.2016–28.02.2020)	COVID-19 Era (01.03.2020–28.02.2022)	*p*-Value
Infection at index bleeding (%)	58 (23.5)	36 (23.8)	22 (22.9)	0.867
Antibiotic prophylaxis (%)	154 (84.6)	89 (81.7)	65 (89.0)	0.176
Intubation during EGD (%)	39 (16.0)	24 (16.1)	15 (16.0)	1.000
Prophylactic intubation	28 (11.5)	19 (12.8)	9 (9.5)	0.538
Infection after bleeding (%)	73 (30.3)	39 (26.5)	34 (36.2)	0.112
Type of infection after bleeding (%)				
Pneumonia	27 (37.0)	18 (46.2)	9 (26.5)	0.082
Spontaneous bacterial peritonitis	11 (15.1)	5 (12.8)	6 (17.6)	0.565
Urinary tract	3 (4.1)	2 (5.1)	1 (2.9)	0.639
Primary bacteremia	11 (15.1)	7 (17.9)	4 (11.8)	0.461
Soft tissue	3 (4.1)	1 (2.6)	2 (5.9)	0.476
Proven bacterial pathogen (%)	42 (17.3)	24 (16.1)	18 (19.1)	0.602
Proven MDR (%)	17 (6.9)	11 (7.3)	6 (6.3)	0.803

Abbreviations: EGD esophagogastroduodenoscopy; MDR: multi-drug resistance.

## Data Availability

Data are available on reasonable request from the corresponding author.
